# Co-axial acoustic-based optical coherence vibrometry probe for the quantification of resonance frequency modes in ocular tissue

**DOI:** 10.1038/s41598-022-21978-8

**Published:** 2022-11-06

**Authors:** Ryan McAuley, A. Nolan, A. Curatolo, S. Alexandrov, F. Zvietcovich, A. Varea Bejar, S. Marcos, M. Leahy, J. S. Birkenfeld

**Affiliations:** 1 Tissue Optics and Microcirculation Imaging Facility, School of Physics, University of Galway, Galway, Ireland; 2 Instituto de Óptica, Consejo Superior de Investigaciones Científicas (IO-CSIC), Madrid, Spain; 3 Institute of Physical Chemistry, Polish Academy of Sciences, Warsaw, Poland; 4 International Centre for Translational Eye Research, Warsaw, Poland; 5 Center for Visual Science, The Institute of Optics, Flaum Eye Institute, University of Rochester, Rochester, New York USA

**Keywords:** Biomedical engineering, Eye diseases, Biophotonics, Imaging and sensing

## Abstract

We present a co-axial acoustic-based optical coherence vibrometry probe (CoA-OCV) for vibro-acoustic resonance quantification in biological tissues. Sample vibrations were stimulated via a loudspeaker, and pre-compensation was used to calibrate the acoustic spectrum. Sample vibrations were measured via phase-sensitive swept-source optical coherence tomography (OCT). Resonance frequencies of corneal phantoms were measured at varying intraocular pressures (IOP), and dependencies on Young´s Modulus (E), phantom thickness and IOP were observed. Cycling IOP revealed hysteresis. For E = 0.3 MPa, resonance frequencies increased with IOP at a rate of 3.9, 3.7 and 3.5 Hz/mmHg for varied thicknesses and 1.7, 2.5 and 2.8 Hz/mmHg for E = 0.16 MPa. Resonance frequencies increased with thickness at a rate of 0.25 Hz/µm for E = 0.3 MPa, and 0.40 Hz/µm for E = 0.16 MPa. E showed the most predominant impact in the shift of the resonance frequencies. Full width at half maximum (FWHM) of the resonance modes increased with increasing thickness and decreased with increasing E. Only thickness and E contributed to the variance of FWHM. In rabbit corneas, resonance frequencies of 360–460 Hz were observed. The results of the current study demonstrate the feasibility of CoA-OCV for use in future OCT-V studies.

## Introduction

The mechanical properties of biological tissues play an essential role in maintaining the structural stability and adequate function of organs in the human body. Abnormal biomechanics in living tissues can be an indicator of pathologies or injury, and as such, the measurement and quantification of biomechanical properties of tissue is relevant to support early diagnosis of diseases^1–4^. A considerable amount of recent research effort has been focused on the development of measurement techniques for the quantification of biomechanics in ocular tissues, particularly in the cornea^1,3,5–15^ although, in recent years, a number of techniques were adapted for the estimation of biomechanics in the crystalline lens (e.g.^16–18^) and the sclera (e.g.^6,19–23^). Corneal biomechanics contribute significantly to the maintaining of adequate corneal shape and function, therefore, abnormal biomechanical properties can be an indicator of corneal pathologies or structural deterioration^1,12,24,25^. Keratoconus (KC), an ocular pathology characterised by corneal thinning and steepening, is hypothesized to be preceded by a localized biomechanical weakening of the corneal tissue^26^. Some reports associate iatrogenically induced ectasia after LASIK surgery in some patients to abnormal corneal biomechanics^12,24,27,28^. As such, detection of biomechanical abnormalities can be valuable not only in the early diagnosis of corneal pathologies like KC, but also, in combination with computational models, to predict the outcome of interventional procedures determining the likelihood of success and helping to identify patients at risk of developing iatrogenic ectasia^3,12,29,30^.

There exists a number of techniques for the assessment of corneal tissue biomechanics such as, Ultrasound Elastography (USE)^31,32^, Magnetic Resonance Elastography (MRE)^33^, Brillouin microscopy^8,34^, air-puff deformation imaging^35–37^, and Optical Coherence Elastography (OCE)^1,3–5,34^. OCE, an optical technique which is applicable in vivo, is extensively used in corneal biomechanics research owing to its superior resolution, sensitivity and potentially non-contact nature^1,5,25,38^. OCE measures the mechanical response of tissues to external stimuli such as ultrasound excitation or air-puff on a multitude of spatial scales depending on application. Dynamic tissue responses can be assessed through OCE measurements in a number of ways such as elastic wave propagation velocities^5,9^ and natural or resonance frequencies^1,3,39^. These measurements allow for the differentiation between normal tissues and tissues effected by pathologies or assess the outcome of surgical interventions.

OCT vibrometry/vibrography (OCT-V) has been proposed as an alternative to the techniques above to measure corneal biomechanics. In its current embodiment, the investigated sample is typically stimulated by acoustic waves from a speaker, and phase-sensitive OCT imaging (PhSOCT) is used to measure the resonance amplitudes which are expected to be on a micro and sub-microscale^29,40–43^. OCT-V is innately similar to OCE both in optical measurement scheme, and stimulation techniques, i.e., low frequency acoustic in OCT-V and high frequency acoustic in some iterations of OCE. Both OCE and OCT-V can be used to quantify the natural or resonance frequency of corneal tissue through broadband excitation of vibrational displacements. Indeed, OCT-V may be considered a subset of OCE techniques that is focused on the low frequency acoustic regime of vibrational resonance characterisation of tissues. This is evident when considering techniques such as acoustic radiation force optical coherence elastography (ARF-OCE), where resonance at low frequencies has been measured in phantoms using amplitude modulated, high frequency acoustic stimulation, utilizing an ultrasound carrier frequency^44^. As such, results from OCE and OCT-V can be considered complementary. For instance, a recently developed OCE technique based on natural frequency quantification in corneal tissue stimulated with a low-force highly-focused microliter air-pulse showed a repeatable measurement of natural frequencies of in vivo human corneas in the range of 234 to 277 Hz^39^, while an OCT-V study based on acoustic stimulation from a speaker beneath an ex vivo human eye demonstrated corneal resonance frequencies of 130 (at 7 mmHg) to 140 Hz (50 mmHg)^42^.

Corneal resonance modes are thought to provide information about the cornea due to their sensitivity to biomechanics^29,40^, analogous to the resonance modes of a thin stretched elastic membrane, the frequency of which are sensitive to the membrane’s properties such as thickness, radius, E and in-plane tension. For example, Akca et al., demonstrated that the first three radially symmetric resonance modes of the corneas of ex vivo bovine eye globes showed a positive correlation between weight and age of the eyes, and the corneal resonance frequencies^29^. In another study, OCT-V experiments using corneal collagen crosslinking (CXL)^45^ for corneal stiffening, demonstrated that the resonance mode frequencies of ex vivo bovine and porcine corneal flaps increased with increasing corneal stiffness^40^. These experiments also showed that a similar sensitivity of the resonance modes to stiffness, induced by CXL, can be seen in both ex vivo corneal buttons and the corneas of whole eye globes. The latter study was accompanied by finite element modelling (FEM), simulating corneal resonance frequency characteristics with changes in elasticity, density, thickness, diameter, IOP and curvature. The results from FEM suggested that corneal vibrations in whole eye globes are primarily sensitive to corneal biomechanical parameters. While it seems there is some agreement in the literature between experimental results and simulations in regard to the elasticity i.e. increased E of the cornea results in an increased resonance mode frequency, there are discrepancies on the impact of ocular properties such as corneal thickness and IOP on the corneal resonance frequency. This confounds the development of OCT-V in certain contexts by suggesting that OCT-V measurements may indeed be insensitive to particular ocular parameters under varied conditions. For instance, there appears to be no consensus in the literature as to whether IOP changes result in a significant shift in the resonance frequency of the cornea or if these resonance modes are actually insensitive to IOP^40,46–50^. While inconsistencies between results of various papers may be attributed to the complex nature and structure of the eye and cornea which will differ between investigated sample and measurement context, it is important that results are aligned, and the origins of differences are understood as to not hinder the development of OCT-V for corneal biomechanics quantification.

Along with a lack of consensus in the literature as to the influence of ocular properties such as IOP on the resonance modes of the cornea, there is also a noticeable lack of consideration in some OCT-V studies for the angle of the acoustic stimulation module relative to surface of the sample under investigation^29,40,41^. The speaker in these experiments would appear to be positioned at an arbitrary (or not described) angle with reference to the sample, although one study does mention adjusting the orientation of the speaker to observe the effect on the resonance modes^29^. This experimental intricacy is important as it is understood that the acoustic absorption and transmission coefficients of a membrane will have different values depending on the incident angle of the waves to the sample surface: as the angle of incidence deviates from normal to the sample (90°), the displacement amplitudes of a membrane in response to a harmonic acoustic stimulation will decrease. Furthermore, coupling of acoustic waves at a given frequency also varies with angle, introducing further complexity to resonance frequency measurements. This is a particular problem in samples which have a broader resonance peak like the cornea, since the resonance mode could be misidentified, identified at the wrong frequency, or the FWHM could be incorrectly quantified due to angle-induced variation in the measured frequency response. However, it is understood that the necessity of an angle in the incidence of the acoustic stimulation arises from the speaker impeding the field of view of the OCT system, thus the speaker is typically positioned obliquely to the optical imaging axis to allow simultaneous acoustic stimulation and measurement of sample displacement. Recent OCT-V studies on in vitro corneal tissues have incorporated acoustic stimulation co-linearly with the imaging axis of OCT system, at 90° to the sample, but the speaker was positioned below the sample with stimulation output facing the OCT objective lens i.e., the tissue was positioned between the OCT system imaging lens and the speaker^42^. While this configuration does eliminate the need for angled coupling of the acoustic waves and the sample it does omit this setup from in vivo measurements.

In OCT-V, the acoustic waves incident on the sample are produced by a speaker which is being fed a voltage waveform signal. Typical loudspeakers exhibit an acoustic frequency response, so it is expected that the relative amplitudes of each frequency component of the acoustic waves produced will vary. To ensure consistent measurements, the speaker’s frequency response needs to be characterized and accounted for as any peaks or gradients in the frequency response will invariably have an effect on that measured from the sample. Additionally, because of the attenuation of acoustic waves at different frequencies in air, and interference due to reflections in the space in which the speaker is enclosed, the acoustic frequency content incident on the sample is expected to vary considerably. In an earlier OCT-V study^29^, the authors measured the sound pressure level (SPL) of all frequency components from a speaker, and then using this calibration data, the frequency responses measured from the cornea were adjusted, but a detailed description of the measurement method is missing. Assuming a microphone was used, the orientation and position of the microphone relative to the speaker during calibration is important as typical microphones have some directivity and so the frequency response will vary with angle. Calibration has also been described by measuring the frequency response of the speaker membrane movement using a spectral domain OCT system^42,51^. However, the frequency response of the speaker membrane movement may differ from the spectrum of the produced acoustic waves. Therefore the calibration spectrum derived from speaker movement could introduce further errors in the results, unless the speaker was driving oscillations in the tissue by physically touching the tissue.

In this work, we present a non-invasive co-axial acoustic-based optical coherence vibrometry probe (CoA-OCV) to meet the above described challenges in OCT-V and support effective use in corneal biomechanics research and studies of diseases which affect tissue biomechanics. More specifically, our approach reduces the most prevalent confounding factors in current OCT-V setups, namely the angled coupling of acoustics waves and sample, and inadequate compensation of acoustic stimulation frequency content at the sample. CoA-OCV includes, for the first time to our knowledge, a co-axial alignment of the speaker, OCT system and sample which ensures uniform acoustic stimulation of the corneal vibrations, i.e. it permits for acoustic waves to propagate at normal incidence to the apex of the cornea with simultaneous displacement measurement from the OCT system. In addition, we have developed a customized pre-compensation process that is used to quantify and compensate for the frequency content of the acoustic waves at the surface of the cornea. The pre-compensation of the acoustic stimulation frequency content and construction of acoustic signals in the Fourier domain allowed for calibration of the acoustic signals and suppression of Fresnel ripple due to linear frequency sweeping.

We have demonstrated the efficacy of the CoA-OCV probe on hydrophilic corneal phantoms (CPs) of varied E, thickness and IOP. The measurement of CPs provided viscoelastic models of the cornea and help set a baseline expectation as to how the cornea will behave under similar conditions. The CP study was followed up by a proof-of-concept study using ex vivo rabbit corneas for a first estimation on the potential applicability of CoA-OCV in vivo. Our results suggest the feasibility of CoA-OCV not only for the cornea, but possibly for other tissues, such as the skin and tympanic membrane. Furthermore, this coaxial approach of the speaker and OCT imaging system could also be utilised in general OCE as well, where other coaxial approaches involving ultrasound ring transducers^52^, confocal air-coupled ultrasound^9^ and mechanical ring actuators^53^ have been proposed.

## Methods

In this section, the OCT imaging system used for resonance frequency measurements is described and the co-axial alignment of the speaker and OCT beam configuration introduced. The acoustic pre-compensation methodology is also introduced and scanning protocol and data analysis described.

### OCT imaging system

**Figure 1:**
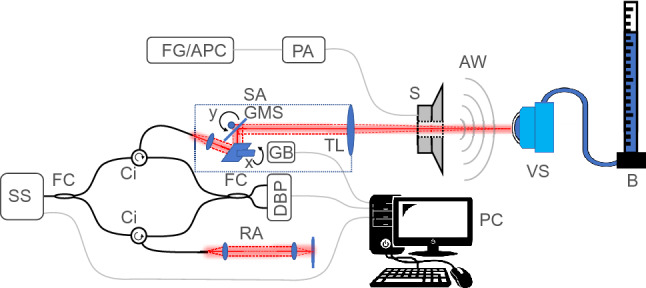
Schematic of CoA-OCV system with co-axial speaker and imaging alignment. (SS)—swept source laser, (FC)—fibre couplers, (Ci)—circulators, (RA)—reference arm, (DBP)—dual balanced photodetector, (PC)—controlling computer, (GB)—galvanometer digital processing board, (GMS)—galvanometer mirror system, (TL)—telecentric f-theta lens, (S)—speaker, (AW)—acoustic waves, (SA)—sample arm, (VS)—vibrometry sample, (B)—barometer, (PA)—power amplifier, and (FG/APC)—function generator or audio controlling computer. The imaging system, the speaker, and the sample are separately mounted on individual mechanical stages to avoid displacements in the sample stimulated through vibrational coupling of the components. Adapted with permission from^36^ © The Optical Society.

A custom-built swept-source OCT (SS-OCT) system^36^ was used to measure corneal displacements at the apex in response to acoustic stimulation from a speaker. A schematic for the system is shown in Fig. 1. The OCT system architecture includes a Mach-Zender type interferometer with a swept source laser (SL132120, Thorlabs, USA) for illumination, a telecentric scan lens (LSM05, Thorlabs, USA) for focusing and collection of backscattered light from samples, a dual balanced photodetector (PDB480C-AC, Thorlabs, USA) for detection of interference and a 12-bit digitizer (ATS 9360, Alazartech, Canada) for digitization of the interference signal. Lateral scanning (x, y) of the illuminating beam was achieved with a galvanometer mirror system consisting of two galvanometric scanning mirrors (Saturn 1B, ScannerMAX, Pangolin, USA) which were controlled and synchronised for scanning with an analogue input/output device (PCI-6731, National Instrument, USA). The central wavelength of the swept source laser is 1300 nm with a 3-dB bandwidth of 50 nm and the system axial and lateral resolution are 16 µm and 40 µm (at focus), respectively with an axial depth range of 16 mm in air and 1920 pixels per A-line, when the acquisition of each interferogram datapoint is triggered, on both the rising and falling edges, by an external k-clock signal as outputted by the light source.

The OCT system was operated in motion mode (M-mode) to achieve A-line scanning at a single lateral position over time at a rate of 200 kHz, allowing a temporal sampling period of 5 µs. The vibrations of the sample were acoustically stimulated by a speaker and measured with the OCT system simultaneously over a period of 1 s. The PhSOCT approach was used for the extraction of sample displacement over time from the recorded spectral interferograms. The experimentally-measured phase stability of the SS-OCT system limits the displacement sensitivity between consecutive A-lines to 0.3 nm when operated in M-mode which is sufficient for the measurement of mechanical vibrations of the samples in this study, which are expected to have amplitudes in the range of nanometres to micrometres^29,40–43^. The distance from the objective lens of the system and the sample surface was 110 mm and the speaker was positioned co-axially with the imaging beam at a distance of approximately 7.5 mm from the sample surface.

### Co-axial acoustic stimulation

**Figure 2:**
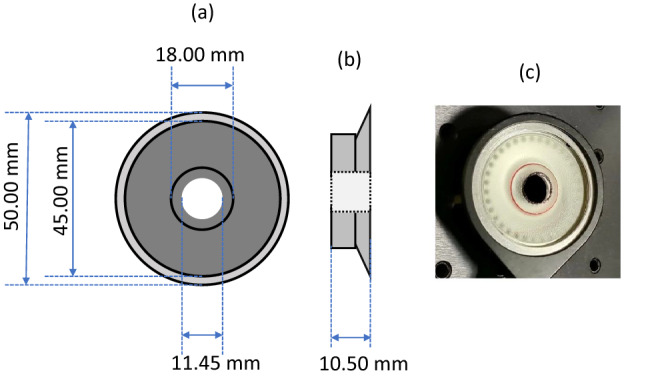
(**a**) Front view of speaker with dimensions, (**b**) side view of speaker with thickness, (**c**) image of speaker in optical mount.

Acoustic chirp waveforms of a specified bandwidth (see “Acoustic pre-compensation” section) from a speaker were directed perpendicularly to the sample surface at the apex of curvature to stimulate the mechanical vibrational displacements of the investigated samples. The speaker used in the setup was a headphone speaker with an architecture based on Tesla Technology (Beyerdynamic GmbH & Co. KG, Heilbronn, Germany). It has an outer diameter of 45.0 mm and thickness of 10.5 mm and has a ventilation port (a hole through the central components of the speaker) with a diameter of 11.5 mm (see Fig. 2a,b). The specified impedance of the speaker is 250 Ω (AC) with a sensitivity of 103 ± 2 dB (at 1 kHz). The speaker was adjusted by opening a 10.0 mm diameter circular aperture at the centre of the membrane (cone) which was aligned with the ventilation port, as to allow the OCT imaging beam to pass fully through the speaker (see Fig. 1). Figure 2a–c show a front view of the speaker with a central, circular aperture, a side view of the speaker and an image of the speaker respectively.

This setup allowed for three important aspects of resonance frequency measurement, namely a) the OCT imaging beam could pass through the speaker onto the sample and reflect back through the speaker to the detector, unimpeded, b) sample alignment was facilitated through a circular, lateral field of view of about 10.0 mm diameter, and c) the speaker could be co-axially aligned with the OCT beam with normal incidence of the acoustic waves to the sample surface. This co-axial alignment of speaker and OCT beam allows to discard the dependence of acoustic stimulation frequency amplitude of the samples on angle of incidence and the signal amplitude in general with angle of incidence in the low frequency acoustic vibrometry regime, as the stimulation profile has a central symmetry about the optical axis of the eye at its apex.

### Acoustic pre-compensation

**Figure 3:**
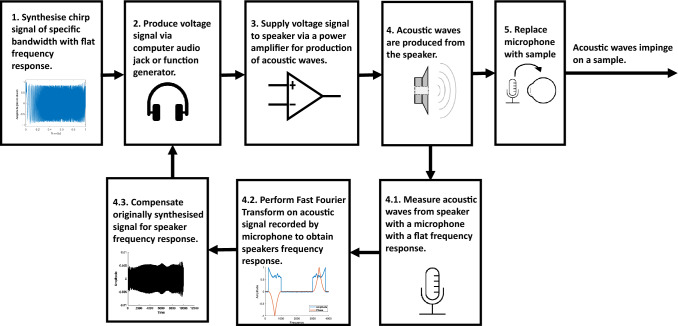
Block diagram describing the process by which the frequency content of the acoustic waves from the speaker can be compensated for. A detailed description of each block can be found in the supplementary information.

A pre-compensation methodology was followed to account for the speaker’s frequency response (due to the coupled speaker components) and the possible geometric and environmental influences on the frequency content of acoustic waves impinging on the sample. This pre-compensation process enabled the synthesis of an acoustic linear frequency chirp with a flat frequency bandwidth to stimulate sample vibrations, compensating for variations in the acoustic frequency spectrum other than from the sample. The block diagram in Fig. 3 illustrates the developed pre-compensation process in a step-by-step manner and detailed descriptions of each block can be found in supplementary information of this study. In short, this process involves the synthesis of an acoustic linear frequency chirp with suppressed Fresnel ripple (similar to a Schröder multisine approach^54^), output and amplification of the chirp signal as a voltage to the speaker, measurement of the acoustic content at the location of the sample with a microphone, retrieval of the frequency spectrum of the acoustic stimulation via fast Fourier transform (FFT) and spectral shaping of the frequency content of the chirp to account for variations in the frequency spectrum.

The frequency content of the signal was measured with an omnidirectional electret microphone (part no. 7717014P, RS Components, United Kingdom), which had a flat frequency response over the frequency range of interest, was oriented with its input facing normal to the speaker. The flat frequency response of the microphone serves as to not affect the frequency amplitude spectrum of the measured acoustic waves from the speaker. The microphone had an outer diameter of 6.0 mm and a sensitivity of − 42 ± 3 dB (at 1 kHz). Once pre-compensation was completed, the microphone was removed from in front of the speaker, and the sample of interest situated facing the speaker. The sample, speaker and SS-OCT were all situated on different mounts/tables as to neglect vibrational stimulation of the sample and OCT system by the speaker through any other means than acoustically through air.

### Data processing and analysis

All M-mode measurements consisted of 200,000 A-lines acquired over a 1 s duration at a lateral position corresponding to the apex of the CPs and rabbit corneas. For each A-line, the acquired k-clocked interferogram was numerically pre-processed, including DC subtraction, and apodization (spectral shaping). Then, the Inverse Fast Fourier Transform (IFFT) of each processed interferogram was computed to generate complex-valued A-lines, encoding the depth profile of the sample-induced backscattered light irradiance in the absolute value information.

Displacements of the samples in response to the acoustic stimulation were extracted from the M-mode phase component of the A-lines, following the PhSOCT approach. The phase information for all pixels in the M-mode scan were retrieved from the complex-valued output of the IFFTs by computation of the four-quadrant inverse tangent of each complex number (each corresponding to a pixel in depth). Subsequently, a pixel was selected from the first A-line which would correspond to the surface of the sample, typically the absolute value (OCT amplitude) corresponding to this pixel was relatively high. The phase for this pixel was then tracked between all adjacent A-lines in the M-mode scan, the resultant signal corresponding to the phase variation due to displacement of the sample over time. The phase differences were then retrieved by subtracting the phases between adjacent A-lines. The phase differences were then converted to spatial displacements via the following equation^55^.

$$\Delta z = \lambda_{0} {\raise0.7ex\hbox{${\Delta \theta }$} \!\mathord{\left/ {\vphantom {{\Delta \theta } {4\pi n}}}\right.\kern-\nulldelimiterspace} \!\lower0.7ex\hbox{${4\pi n}$}}$$

where is the *Δz* is the displacement, *λ*_*0*_ is the central wavelength of the illumination source of the SS-OCT system (1300 nm), *Δθ* is the phase difference and *n* is the refractive index, which is approximated as 1 in air in this study.

The resonance frequency was determined from the FFT of Δz over a time interval corresponding to the response of the sample to a full chirp signal. The FFT of the time-dependent response represents the frequency response of the cornea over the bandwidth of stimulation of the chirp, similar to a pulse/delta excitation. The resonance frequency was identified as the frequency with maximum amplitude, after FFT, over the frequency range of stimulation. This amplitude was then used for normalization of the frequency response.

### Validation experiments

To demonstrate the validity of CoA-OCV with the presented pre-compensation and co-axial alignment, various samples were imaged, and their frequency responses obtained. These samples included a tuning fork, artificial corneal phantoms and corneas of ex vivo rabbit eye globes.

#### Tuning fork

After pre-compensation, a tuning fork with a frequency response peak at 440 Hz, was positioned with the end region of its tines (the region of expected maximum displacement) facing the OCT beam. A one second duration band-limited (200–500 Hz) pre-compensated acoustic chirp was played on loop from the speaker and the SS-OCT system recorded 200,000 A-lines over 1 s during the looped chirps. The frequency spectrum of the tuning fork vibrational amplitude time trace was then acquired by FFT. The post holding the tuning fork was also measured to ensure there were no influences from movement other than from the tuning fork.

#### Artificial cornea phantoms

Corneal phantoms (CP) constructed from hydrophilic contact lens material were studied to model the frequency response of the cornea. The CPs consisted of two types (*Gentle* and *Saphir*, manufactured by *Mark'ennovy*, Madrid, Spain), with different material properties and three thicknesses of each type, for a total of six phantoms. Material properties can be seen in Table. 1. The Young’s Modulus was E = 0.16 MPa for CP *Gentle* (G) and E = 0.3 MPa for CP *Saphir* (S). The E and other physical properties of the CPs can be found in earlier literature^30^. Both CP types were manufactured with three uniform thicknesses: 350 μm, 450 μm and 550 μm. Before the measurements, CPs were mounted in an artificial eye chamber (Barron Artificial Chamber, Katena Products Inc, Parsippany, NJ, USA), filled with saline solution (sodium chloride 0.9%), which kept the CP upright and static. CPs were pressurized by a water column system to model predefined changes in IOP. The variation between the elastic properties of the CPs, thicknesses and the ability to simulate IOP allowed to study the dependence of vibrational resonance mode changes with these parameters.

**Table 1:** Material properties of corneal phantoms, adapted from^30^.

Short name	Gentle (G)	Saphir (S)
Brand name	Gentle 80 Ori:gen	Saphir RX Spheric
Material composition	Acrylic Co-Acrylamide Ter-Polymer	Silicone hydrogel
ISO Hydrogel name	Filcon II 3	Filcon V 3
Water content	79% ± 2	75% ± 1
Young’s Modules [MPa]	0.16	0.3
Elongation at break [%]	221	269
https://doi.org/10.1371/journal.pone.0165669.t001		

For all measurements, the CPs were positioned in the OCT imaging range, with their apex approximately 0.5 cm from the speaker. For signal-to-noise ratio (SNR), energy deposition purposes and scanning constraints, a 0.25 s chirp of 200 Hz bandwidth was pre-compensated and used for stimulation. An initial stimulation range of 200–400 Hz was chosen as it has been shown in previous OCT-V experiments and simulations that resonance modes of corneal tissues with similar material properties to the CPs can be observed below 400 Hz^29,40–42,56^.The stimulation range also needed to be above 200 Hz as the speaker used became unstable for frequencies below this. The chirp signal was played on loop while the SS-OCT system recorded 200,000 A-lines over 1 s at the above-described position. Measurements were taken at IOPs cycled between 5 and 25 mmHg in steps of 2.5 mmHg. In between each measurement, the CPs were hydrated with saline solution to maintain hydration. The apex of the CPs was realigned with the OCT beam after every IOP adjustment to compensate for bulging. The frequency bandwidth of stimulation was also adjusted for each CP as to adequately capture the resonance frequency shifts over the IOP range. For example, the bandwidth of acoustic stimulation for the S CP with a thickness of 550 μm was 250–450 Hz and resonance frequencies measured from 334 to 406 Hz, this range was not suitable for the G CP with thickness of 350 μm where the range of measured resonance frequencies was 212–256 Hz and so, a bandwidth of 200–400 Hz was used instead. Besides the resonance frequency, the FWHM of each resonance mode were also quantified.

After data collection, the impact of each variable (IOP, CP thickness, and E) on the movement of the resonance frequency peak was quantified with an analysis of variance (ANOVA) by determining the variance (sum of squares) attributed to each variable after performing a linear regression. The percentage of the total sum of squares for each variable was calculated to compare relative contributions, including the error of the model, to the total variance of the response.

#### Ex vivo rabbit cornea

**Figure 4:**
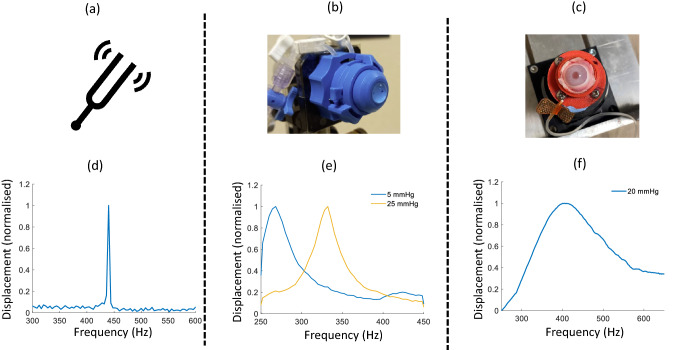
(**a**) Image of tuning, (**d**) Frequency response of the tuning fork. (**b**) is an image of one of the CPs in the custom holder, (**e**) Example frequency responses of CP with E of 0.16 at IOPs of 5 mmHg (blue) and 25 mmHg (yellow), (**c**) Image of a rabbit eye in the custom printed holder without the cap and (**f**) Example frequency response for a rabbit cornea at an IOP of 20 mmHg.

Two eyes were obtained from two adult albino New Zealand rabbits weighing between 2.5 and 3.0 kg and housed in the animal facilities at the University of Valladolid. The treatment protocols were approved by the Animal Ethics Committee at the University of Valladolid. All experiments were performed in accordance with relevant guidelines and regulations. The eyes were kept at approximately 4 °C before sample preparation and all measurements were performed within 48 h post-mortem. Before measurements, the orbital fat was removed, and the eyes were placed in a customized eye holder^36^ and connected to an IOP control system. This allowed a needle to be pierced through the optical nerve into the posterior chamber. The needle was not taken out until completing all the measurements, assuring that the IOP remained constant during the experiments and in between each measurement, the corneas were hydrated with saline solution. The frequency response of the rabbit eyes were measured at three different IOPs (20 mmHg, 25 mmHg and 30 mmHg). The corneal apex was positioned similarly to the CPs (Fig. 4) with the apex approximately 0.5 cm from the speaker. An initial stimulation range of 250–450 Hz was chosen but preliminary measurements showed that the resonance modes of the rabbit cornea were much broader and needed a larger stimulation bandwidth, therefore, a one second chirp signal of larger bandwidth, 250–650 Hz, was played on loop while the SS-OCT system recorded 200,000 A-lines over one second at the measurement point of the cornea. In comparison to the CPs, a lower magnitude SNR was observed in the vibrational displacements of the rabbit corneas. This was due to the fact that the vibrational response of the rabbit corneas to acoustic excitation are lower in amplitude than that of the CPs at the same SPL while the displacement noise is at approximately the same amplitude for both the corneas and CPs. Furthermore, processing and analysis of the cornea frequency responses included shifting mean and median filtering of the data in the Fourier domain to reduce the impact of noise on resonance mode identification.

## Results

### System validation

**Figure 5:**
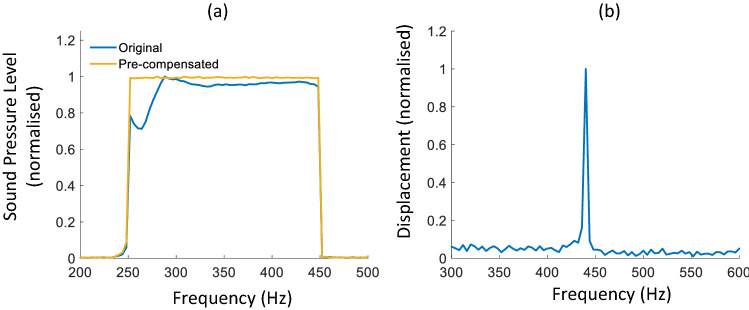
(**a**) Sound pressure level recorded from the microphone before pre-compensation (blue) and after pre-compensation (yellow), (**b**) measured frequency response of a 440 Hz tuning fork.

Figure 5a shows the effect of pre-compensation on the frequency content of the acoustic stimulation: the blue line represents the frequency content of the acoustic signal at the location of the microphone before pre-compensation and the orange line after pre-compensation. After pre-compensation, the frequency content of the signal shows a flat frequency amplitude bandwidth over the range 250–450 Hz. Figure 5b shows the validation of the pre-compensation technique and vibrometry setup using a tuning fork. A clear peak at 440 Hz with an amplitude of approximately 8.7 nm is observed. This result adds validity to the CoA-OCV setup and pre-compensation by adequately acquiring the frequency response of the tuning fork (albeit with some noise).

### Artificial cornea phantoms

**Figure 6:**
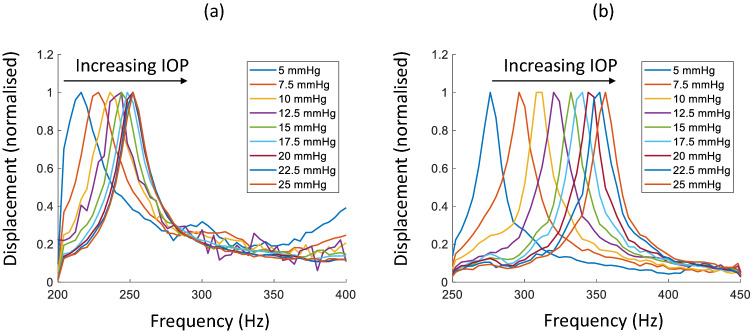
First resonance modes of (**a**) G and (**b**) S with 350 µm thickness for IOPs ranging from 5 to 25 mmHg.

Figure 6 shows the normalised frequency responses of corneal phantoms G (a) and S (b) of constant 350 μm thickness for different IOPs. Resonance peaks are presented from 216 to 252 Hz for G and 276 to 356 Hz for S. The frequency shift for the total 20 mmHg IOP increase for G is 40 Hz and for S, 80 Hz.

**Figure 7:**
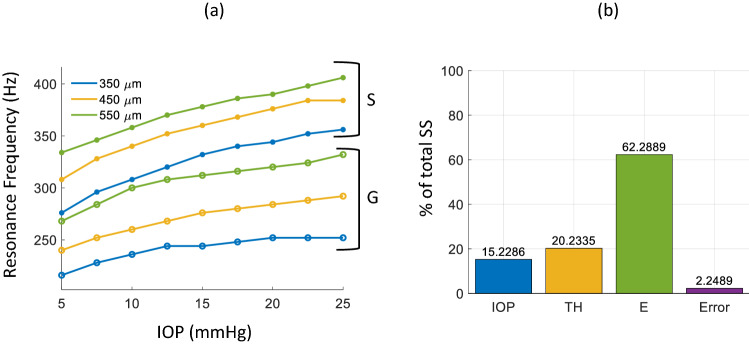
(**a**) IOP vs First resonance peak frequency for S (·) and G (o) with phantom thicknesses of 350 µm (blue), 450 µm (yellow) and 550 µm (green), (**b**) percentage of the total sum of squares of the parameters, intraocular pressure (IOP), CP thickness (TH), and Young´s modulus (E), to the variation of resonance frequency. The percentage of the total sum of squares of the linear regression error is also shown.

Figure 7a shows the change in resonance frequency with IOP for G and S CPs of different thicknesses with increasing IOP. If approximated as a linear fit (shown in supplementary information, Fig. S5), the slope of frequency versus IOP against thickness, was 3.9, 3.7 and 3.5 Hz/mmHg for S, and 1.7, 2.5 and 2.8 Hz/mmHg for G at thickness of 350 µm, 450 µm and 550 µm, respectively. A linear fit of thickness against resonance frequency yielded slopes of 0.25 Hz/µm for S, and 0.33 Hz/µm for G, averaged across IOP conditions (see supplementary information, Fig. S6).

Figure 7b shows the results of the ANOVA for the resonance frequency peak evaluated with the percentage of the total sum of squares (SS) of each parameter (intraocular pressure—IOP, CP thickness—TH, and Young´s modulus—E). All three parameters contributed to the variance of the resonance frequency peak: IOP (15.23%), TH (20.23%), and E (68.29%), all being greater than the model error contribution (2.25%). Moreover, E had the most predominant impact in the movement of the resonance frequency peak.

**Figure 8:**
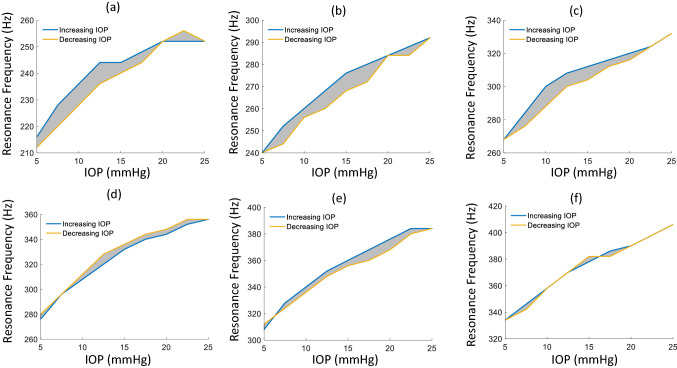
Hysteresis curves for IOP vs CP Peak Resonance frequency for thicknesses 350 µm (**a**), 450 µm (**b**) and 550 µm (**c**) of G and 350 µm (**d**), 450 µm (**e**) and 550 µm (**f**) of S. Blue lines indicate increasing IOP and yellow lines, decreasing IOP.

Figure 8 presents the results of cycling the IOP from lowest to highest values and back. Each CP exhibited some degree of hysteresis during resonance frequency measurement with the cycling of IOP. The degree of hysteresis, calculated by the discrete sum of the shaded areas, were 75, 100, 110 Hz × mmHg for G (Fig. 8a–c) and 75, 85, and 10 Hz × mmHg for S (Fig. 8d–f) at thicknesses of 350, 450 and 550 µm respectively. On average, the degree of hysteresis increased with lens thickness, except for S at 550 µm.

**Figure 9:**
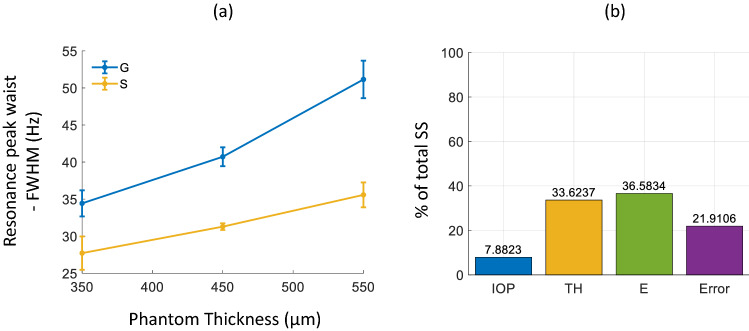
(**a**) FWHM of the resonance mode peaks for corneal phantoms (CPs) of two materials G (E = 0.16 MPa, blue) and S (E = 0.3 MPa, yellow) as a function of thickness. FWHM values were averaged over all IOPs at given thickness for each CP. Error bars show the standard deviation of the FWHM over all IOPs. (**b**) percentage of the total sum of squares of the parameters: intraocular pressure (IOP), CP thickness (TH), and Young´s modulus (E) to the variation of resonance peak waist (measured as the FWHM). The percentage of the total sum of squares of the lineal regression error is also shown.

Figure 9a presents the FWHM of the resonance mode peaks for all 6 CPs. No systematic change was observed for the FWHM values for different IOPs and so the FWHM for each CP at a given thickness was averaged over all measured IOPs. The FWHM was observed to increase with increased thickness and decrease with increased E. FWHM for CPs with E of 0.16 MPa were calculated at 34, 41 and 51 Hz for thicknesses of 350, 450 and 550 µm, respectively. FWHM for CPs with E of 0.30 MPa were calculated at 28, 31 and 36 Hz for thicknesses of 350, 450 and 550 µm, respectively.

Figure 9b shows the results of the ANOVA for the FWHM evaluated with the percentage of the total SS of investigated parameters. Only two parameters contributed to the variance of the FWHM: TH (33.62%) and E (36.58%) being greater than the error model contribution (21.91%). In this case, IOP does not have a significant impact towards the variance of the resonance peak waist (7.88%).

### Rabbit corneas ex vivo

**Figure 10:**
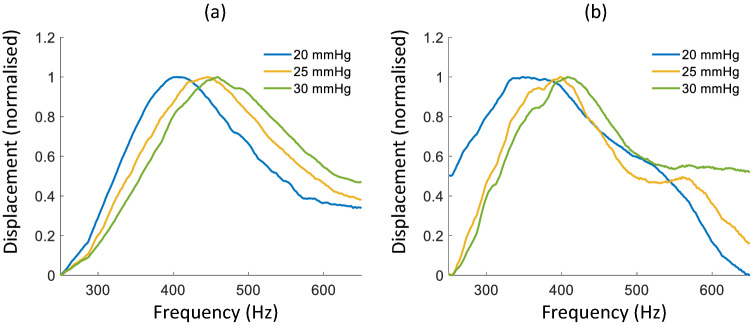
Frequency response of rabbit cornea (**a**, eye 1) and (**b**, eye 2) with normalised amplitude for IOPs of 20 (blue), 25 (yellow) and 30 (green) mmHg.

Figure 10a, b show the measured frequency responses of the two ex vivo rabbit corneas (eye 1, Central Corneal Thickness: 443 µm; eye 2, Central Corneal Thickness: 434 µm) for three different IOPs (20, 25 and 30 mmHg). The resulting displacement curves were visibly broader than those of the CPs and spanned a higher range of frequencies. The resonance frequency shifted towards higher values with increased IOP for both eyes. The resonance peaks for eye 1 were at approximately 405 Hz, 437 Hz and 460 Hz, and for eye 2 at 360 Hz, 385 Hz and 408 Hz (for IOPs 20 mmHg, 25 mmHg and 30 mmHg, respectively). The rate of change of resonance frequency with IOP was 5.5 and 4.8 Hz × mmHg^−1^ for eye 1 and 2, respectively.

## Discussion

In this work we have presented a non-invasive co-axial acoustic-based optical coherence vibrometry (CoA-OCV) probe with a customized pre-compensation process for the use in tissue characterization. CoA-OCV was successfully demonstrated on corneal phantoms and ex vivo rabbit corneas. The controlled study of corneal phantoms of predetermined E and thickness at varying IOPs allowed an estimation of the impact of each parameter to the measured resonance frequency peak and the resonance peak waist. We found that E had the most predominant impact in the movement of the resonance frequency peak, and that IOP does not have a significant impact towards the variance of the resonance peak waist. The observed results provide further support to the use of OCT-V for biomechanical tissue characterization, with potential future use in disease diagnosis. The importance and novelty of the presented work becomes apparent when put in the context of previous OCT-V and tissue biomechanics research where variability of experimental setup and contradictions in results and findings between publications exist.

A recent OCT-V publication focused on the measurement of corneal vibrations with variation in ocular parameters reports an increase in resonance frequency modes and/or damping in peak amplitudes in ex vivo bovine and porcine corneas after CXL, which produces an increase in corneal stiffness^40^. Furthermore, a shift in resonance modes was observed between simulations of a normal eye and an eye with keratoconus. These results are supported by finite-element simulations and are broadly in line with OCE experimental results on agar phantoms, where the natural frequencies of the phantoms were correlated with the square root of the Young’s modulus^57^. Further results from numerical modelling of air-puff induced corneal vibrations indicate that the modelled corneal vibrations are associated with, and suggested to be affected in part, by the corneal elasticity coefficient^58^. The results obtained in the current study showed that for a constant thickness of the corneal phantoms at a fixed IOP, there is an increase in the resonance mode frequency with increased E of the CP materials. In fact, it was found that within the range of thicknesses and IOP of the CPs measured in this study, the frequency of the resonance modes between the CPs was always greater for a higher E at a given thickness and IOP. This suggests a dependence of the resonance mode frequencies on the mechanical properties of the CPs, such as E, and that this technique could have potential clinical use in detecting corneal mechanical abnormalities, for example in KC.

A similar dependence of the resonance modes on the CP thickness could be observed from the results presented in Fig. 7. For a constant IOP and E, an increase of the thickness of the CP results in an increase in the resonance mode frequency. This is however not consistent with all results from previous literature. However, it must be noted that the structure and mechanics of the cornea is much more complex than that of the CPs used. While some publications suggest that the thickness of the cornea does indeed play a role in the corneas natural vibrations, the specific outcome of increased thickness is not clear^29,39,48,59^. For instance, experiments on agar phantoms found that the natural frequency of the phantoms decreased as thickness of the samples increased, this lines up with results from recent FEM research which demonstrated that the resonance modes of a thin walled pre-stressed shell were higher than that of a thick walled counterpart^47,57^. Furthermore, experimental results and numerical simulations found that, for anterior and posterior bovine corneal flaps, an increase in the thickness of the flaps resulted in an increase in the fundamental resonance mode frequency^40,^ which is somewhat in agreement with the results obtained in the current study for the CPs. Complicating the matter further, the same publication also found that the resonance peak frequencies of whole eye globes were nearly independent of corneal thickness.

The contradiction between results from the current and previous studies i.e. whether an increase in the thickness of a measured sample results in an increase or decrease in resonance frequency, may be explained thusly: the increase in resonance mode frequency of the corneal flaps and CPs may not be a direct dependence on the thickness alone but rather a dependence on the changes in other geometrical/mechanical features as a result of varying the thickness, as well as the thickness itself. In an abstract sense, if one is to consider a circular segment of a fluid filled elastic shell with a constant diameter, d, around its periphery, having the displacements/amplitude of vibrations equal to zero at d (similar to boundary conditions in the CPs in this study imposed by the artificial eye chamber with a fixed aperture diameter), under a constant IOP, E and a certain thickness, T, the spherical radius of curvature of the circular segment as it bulges from the boundary condition will have some value, r. The arc length, s, and surface area of this curvature, A, will then depend on r, thus as r increases, so do s and A. If we now consider that r is dependent on the IOP (along with keeping other geometric and mechanical features of the shell segment constant), as IOP increases, r increases and again, so too do s and A (and tension) as a result. Approximating the circular segment of the shell as a flat circular membrane with radius equal to s/2 and tension equal to that of the shell segment, i.e., the surface area of the hypothetical membrane will be equal to A, the rotationally symmetric first resonance mode frequency of such a membrane can be modelled with use of a zeroth order cylindrical Bessel function, scaled to have its first zero at s/2. Subsequently, we then infer the following relationship: the resonance mode frequency of the membrane, described by the aforementioned function, decreases as s increases, analogues to the relationship between a string’s length and its fundamental frequency. Now, if we keep IOP, d and E constant but increase T, it may be observed that s and A will actually decrease due to r decreasing, this being due to the IOP maintaining a smaller r, s and A against the increased thickness of the membrane, possibly resulting in a shift in the resonance mode towards a lower frequency. The implication of this statement is that the spherical curvature and surface area may play a more significant role in the resonance mode frequency than thickness. In reality, the relationship between geometric and mechanical properties of the cornea, such as in-plane tension, curvature, thickness, E and viscoelasticity, hydration etc., and its resonance mode frequencies may be a much more complex interplay of said properties. While parameters such as curvature and thickness were not monitored in this study, monitoring them and analysing further the relationship they have with the resonance modes of the cornea/CPs would be achievable through incorporating M-B mode and M-C mode scanning into the OCT measurement regime, this being a logical next step in our research using the proposed CoA-OCV probe. However, care must be taken in this step as indeed, through the field of view of the cornea which is about 10 mm (limited by the aperture in the speaker), normal incidence is expected to happen only at the apex of the cornea/phantom. The rest of the corneal surface may be at an oblique angle to the acoustic waves which will depend on both the corneal surface normal at any point and the curvature of the sound field. It is not known yet how negligible, or not, this effect maybe; the wavelength of sound may be such that this effect can be ignored. In contrast to acoustic simulations which would be off axis, CoA-OCV allows the cornea to be stimulated uniformly along the circumferential direction and so the acoustic stimulation is axisymmetric and does not depend on the angular position of a measurement point along the corneal surface with reference to the apex. Therefore, there is no side or off-centre region which is acoustically stimulated with a greater/lesser magnitude. This is particularly important with KC where the cone is off centre.

A number of publications have reported via numerical methods, FEM simulations and experiments that the amplitude of resonance modes of the cornea and whole eye globes are sensitive to IOP, some of these purporting the use of corneal resonance mode measurements for use in tonometry^41,49,59^. Similarly, there has been research conducted which has shown development of amplitude-based vibration tonometry, not necessarily at the resonance frequency of the cornea/whole eye^60^. While there would seem to be commonality in publications where resonance mode amplitude and IOP are concerned, it appears that there is no consensus in the literature as to the extent of the affect of IOP on the frequency of corneal resonance modes. FEM simulations found that the frequency of the corneal resonance modes of whole eye globes had only a very minor dependency on IOP, a finding which was supported by a clinical study which demonstrated the lack of correlation between the frequency of corneal modes and IOP^46^. However, other simulations showed that the resonance mode frequencies of whole eyes show a more moderate increase with IOP^46,48^. This relationship being further verified by experimental observation of ex vivo porcine whole eye globe vibrational resonance mode frequencies increasing with IOP and in vivo human air-puff tonometry measurements which showed a high positive correlation between the higher order frequencies induced by the air-puff and the IOP^49,50^. Comparing these findings with that of the current study, in particular the results of Figs. 7 and 10, there indeed does appear to be a positive correlation between the resonance modes of both the CPs and rabbit eyes and their IOP. This, and the previous studies examining relationships between the corneal resonance modes and IOP, help illustrate the role IOP plays in maintaining corneal biomechanics and ocular health, an important endeavour as this lends to the possible use of vibrational tonometry as an alternative to current clinical standard practices for glaucoma testing. However, it must be stated that there are still obstacles to overcome, such as the influence of the many other ocular parameters e.g. viscoelasticity and hydration, on the resonance modes. As such, these obstacles may explain the discrepancies between results of the current and previous studies, although these discrepancies may also be affected by the nature of the experimental methods in each publication, where influences such as corneal tissue type (human, porcine, bovine), simplifications in numerical models, excitation method, etc., play a role.

An extensively studied aspect of corneal dynamics and biomechanics is that of corneal hysteresis. There is a wealth of literature studying the link between corneal hysteresis and glaucoma, keratoconus and tissue aging^61,62^. As such, studying corneal hysteresis with new methods helps support the continuous development of it as a biomarker for corneal pathologies. Indeed there is significant interest in devices for the facilitation of corneal hysteresis measurements such as the Ocular Response Analyzer (Reichert Ophthalmic Instruments, Buffalo, NY)^63^. The viscoelastic characteristics of the cornea are indicated by the measurement of corneal hysteresis and this gives an indication of the cornea’s energy absorption/dissipation properties^37,61^. The viscoelasticity of the cornea is thought to play a role in the vibrational characteristics of the cornea i.e. (time dependant) dampening of the vibrational amplitudes, phase lags etc^58,59^. Figure 8 shows the results of resonance mode measurements of the CPs with cycling IOP up and down. With the exception of the Saphir CP with 550 µm thickness, all CPs demonstrated noticeable hysteresis for the loading and unloading force exerted on the CP by the IOP during the resonance measurements. An interesting finding, these hysteresis loops may be indicative of the differences in corneal radii of curvature invoked by the same IOP and may be considered a measure of viscoelasticity. This makes further sense for these hysteresis loops to occur if we consider the typical method of corneal hysteresis where it is the difference in corneal applanations during loading and unloading of force due to air-puff that is used as a measurement of viscoelasticity^64^. It must be noted however that these results, while only demonstrated on corneal phantoms, are at odds with that from another publication which found no noticeable hysteresis in corneal resonance peak frequency with IOP cycling in freshly enucleated bovine eyeballs^41^.

Corneal viscoelasticity has been suggested in a previous study to be related to the resonance mode peaks in the mechanical frequency response of simulated corneal flaps^40^. Figure 9 shows the FWHM of the resonance modes (averaged over IOP) for the CPs is sensitive to both thickness of the CP and E. As the thickness of the material increases, so does the FWHM but as E is increased, the FWHM decreases. Superficially, this result may suggest that the viscoelastic damping may have a dependence on E, but this does not take into account that the CPs are composed of different materials with differing water contents and so vicious behaviour may be independent of a specific E. Furthermore, the suggestion that the FWHM of corneal resonance modes is dependent on viscoelasticity is somewhat in agreement with the results of the current study if we are to assume that the rabbit cornea has a higher viscosity than the CPs. Also, comparing Figs. 6 and 10, a much broader peak is observed in the rabbit corneas, indicating a higher level of energy dissipation, as assumed in earlier literature^30^. Quantification of the FWHM from results in Fig. 10a yielded an increase in FWHM with IOP (about 45 Hz difference between 20 and 30 mmHg) suggesting viscoelastic damping increases with increased IOP in biological tissue. Corresponding results from Fig. 10b are more ambiguous and the FWHM gives inconclusive results, calling for further investigation.

With so many variable parameters in the cornea, it is imperative to also consider experimental intricacies in OCT-V studies, such as the angle of the speaker relative to the surface of the sample, as these could introduce another level of complexity to corneal resonance mode measurements. An early OCT-V paper which demonstrated the technique used a loudspeaker to acoustically excite the first two resonance modes of a latex membrane stretched over a rigid cylinder and measured the displacements using PhSOCT^43^. The setup schematic in this paper shows the speaker at an oblique angle to the surface membrane as to allow simultaneous stimulation of the resonance modes and measurement with OCT but it is not stated at what angle the speaker is at nor why this was chosen. A more recent paper observed the first three radially symmetric resonance modes of ex vivo bovine cornea with OCT-V, again, stimulated by a speaker at an angle^29^. This paper observed that while the measured resonance frequencies did not change with speaker orientation, the relative amplitudes of the modes did. The widths of the resonance mode peaks however, are not mentioned. It stands to reason that the widths may indeed be affected by the orientation of the speaker which may complicate the analysis of results and hinder measurements of intrinsic properties of the cornea such as viscoelasticity. This is particularly important when analysing samples, such as the rabbit cornea, where a broader and nosier frequency response resonance peak complicates locating the peak frequency further. Other OCT-V publications neglect to mention the orientation of the speaker in their experiments^40,41^. The work in this paper serves to rectify this by the introduction of the co-axial acoustic approach for use in future experiments, neglecting the need for an arbitrary angle of incidence to be chosen. Our proposed setup allows for the acoustic excitation at a normal incidence to the corneal apex. Furthermore, the affect of the speaker angle on the shape of the waveform across the cornea has not yet been studied in detail, nor has the aspect of asymmetric corneal modes. However, a publication studying corneal vibrations with non-contact air-puff tonometry has analysed asymmetric modes induced in the cornea during applanation^50^. In this paper, it is suggested that the prevalence of asymmetric corneal modes may be due to the alignment of the air pump and cornea and/or the geometry of the cornea. This suggestion fits well into the intended future work where the asymmetric corneal modes will be studied using our proposed CoA-OCV probe. Studying asymmetric corneal modes and their dependence on corneal geometry and material properties (global and local) could be a difficult endeavour if the asymmetric modes have a dependence on the angle of the incident stimulation, as suggested in the aforementioned paper. Therefore, CoA-OCV could help in discriminating the corneal parameters affecting asymmetric corneal resonance modes unencumbered by influences of stimulation angle.

The results of the current study demonstrate the applicability of the CoA-OCV probe with customized pre-compensation for use in future OCT-V studies by successfully measuring CPs with different biomechanics, and ex vivo rabbit corneas. It can be inferred from these results that our technique may be suitable for measuring the frequency response of human corneas, and other tissues. The ability of OCT-V to measure the frequency response of the samples with amplitudes at the sub-micron level induced by acoustic waves, in contrast to air-puff based approaches, further supports the possibility of a more patient friendly clinical instrument. And, while measurement of resonance frequency alone could be considered as of limited use, its dependency on biomechanical properties of the cornea and correlation with properties such as E and IOP, can yield a meaningful relationship in a clinical setting. Specifically, taking into account measurable quantities such as corneal thickness and radius of curvature, a numerical model can be built as to predict E for a particular resonance frequency. For a cornea suspected of KC, a low E predicted from the resonance frequency measurement and numerical model would give a clinician a relevant biomechanical quantity. Recent FEM research has shown that the vibrational response of healthy and cross-linked ex vivo corneas can be predicted by numerical modelling which is of great potential for clinical use^40^. However, current models require further development for the in vivo case to include the effect of other properties of the eye (such as that of the ocular muscles) on the resonance frequencies.

## Conclusion

This study demonstrates the successful use of an advanced configuration of OCT-V (CoA-OCV). The co-axial acoustic-based setup with a customized pre-compensation process reduces current confounding factors in OCT-V, namely the angled coupling of acoustics waves and sample, and inadequate compensation of acoustic stimulation frequency content at the sample. In a controlled study using corneal phantoms, the measured resonance modes were found to be sensitive to intrinsic and extrinsic properties of the measured phantoms, such as thickness, Young’s modulus and intraocular pressure. However, the Young´s modulus had the most predominant impact in the movement of the resonance frequency peak. The FWHM of the resonance peaks were only sensitive to thickness, Young’s modulus. A proof-of-concept study showed that CoA-OCV produced measurable resonance frequencies on ex vivo ocular tissue. Future studies will incorporate CoA-OCV into human in vivo studies.

## Supplementary Information

Supplementary Information.

## Data Availability

All data generated or analysed during this study are included in this published article. The datasets generated during and/or analysed during the current study are available from the corresponding author on reasonable request.

## References

[1] Wang S, Larin KV (2015). Optical coherence elastography for tissue characterization: A review. J. Biophoton..

[2] Lee GYH, Lim CT (2007). Biomechanics approaches to studying human diseases. Trends Biotechnol..

[3] Kirby MA (2017). Optical coherence elastography in ophthalmology. J. Biomed. Opt..

[4] Larin KV, Sampson DD (2017). Optical coherence elastography - OCT at work in tissue biomechanics [Invited]. Biomed. Opt. Express.

[5] Zvietcovich F, Larin KV (2022). Wave-based optical coherence elastography: The 10-year perspective. Prog. Biomed. Eng..

[6] Ramier A (2020). In vivo measurement of shear modulus of the human cornea using optical coherence elastography. Sci. Rep..

[7] Nair A, Singh M, Aglyamov S, Larin KV (2021). Heartbeat optical coherence elastography: Corneal biomechanics in vivo’. J. Biomed. Opt..

[8] Scarcelli G, Pineda R, Yun SH (2012). Brillouin optical microscopy for corneal biomechanics. Invest. Ophthalmol. Vis. Sci..

[9] Zvietcovich F (2020). Confocal air-coupled ultrasonic optical coherence elastography probe for quantitative biomechanics. Opt. Lett..

[10] Kling S (2020). Optical coherence elastography by ambient pressure modulation for high-resolution strain mapping applied to patterned cross-linking. J. R. Soc. Interface..

[11] Ramier A, Tavakol B, Yun S-H (2019). Measuring mechanical wave speed, dispersion, and viscoelastic modulus of the cornea using optical coherence elastography. Opt. Express.

[12] Kling S, Hafezi F (2017). Corneal biomechanics – A review. Ophthalmic Physiol. Opt..

[13] Piñero DP, Alcón N (2015). Corneal biomechanics: A review. Clin. Exp. Optom..

[14] Ruberti JW, Sinha Roy A, Roberts CJ (2011). Corneal biomechanics and biomaterials. Annu. Rev. Biomed. Eng..

[15] Lopes BT (2021). Review of in-vivo characterisation of corneal biomechanics. Med. Nov. Technol. Dev..

[16] Wu C, Aglyamov SR, Han Z, Singh M, Liu C-H, Larin KV (2018). Assessing the biomechanical properties of the porcine crystalline lens as a function of intraocular pressure with optical coherence elastography. Biomed. Opt. Express.

[17] Li Y (2019). Simultaneously imaging and quantifying *in vivo* mechanical properties of crystalline lens and cornea using optical coherence elastography with acoustic radiation force excitation. APL Photonics.

[18] Scarcelli G, Kim P, Yun SH (2011). In vivo measurement of age-related stiffening in the crystalline lens by brillouin optical microscopy. Biophys. J ..

[19] Whitford C, Joda A, Jones S, Bao F, Rama P, Elsheikh A (2016). Ex vivo testing of intact eye globes under inflation conditions to determine regional variation of mechanical stiffness. Eye Vis..

[20] Bronte-Ciriza D (2021). Estimation of scleral mechanical properties from air-puff optical coherence tomography. Biomed. Opt. Express.

[21] Elsheikh A, Geraghty B, Alhasso D, Knappett J, Campanelli M, Rama P (2010). Regional variation in the biomechanical properties of the human sclera. Exp. Eye Res..

[22] Wollensak G, Iomdina E (2009). Long-term biomechanical properties of rabbit sclera after collagen crosslinking using riboflavin and ultraviolet A (UVA). Acta Ophthalmol..

[23] Nguyen BA, Roberts CJ, Reilly MA (2019). Biomechanical impact of the sclera on corneal deformation response to an air-puff: a finite-element study. Front. Bioeng. Biotechnol..

[24] Moshirfar M, Behunin N, Christiansen S, Edmonds J (2013). Corneal biomechanics in iatrogenic ectasia and keratoconus: A review of the literature. Oman J. Ophthalmol..

[25] Han Z (2017). Optical coherence elastography assessment of corneal viscoelasticity with a modified Rayleigh-Lamb wave model. J. Mech. Behav. Biomed. Mater..

[26] Roberts CJ, Dupps WJ (2014). Biomechanics of corneal ectasia and biomechanical treatments. J. Cataract Refract. Surg..

[27] Binder PS (2005). Keratoconus and corneal ectasia after LASIK. J. Cataract Refract. Surg..

[28] Wilson A, Marshall J (2020). A review of corneal biomechanics: Mechanisms for measurement and the implications for refractive surgery. Indian J. Ophthalmol..

[29] Akca BI (2015). Observation of sound-induced corneal vibrational modes by optical coherence tomography. Biomed. Opt. Express.

[30] Bekesi N, Dorronsoro C, de la Hoz A, Marcos S (2016). Material properties from air puff corneal deformation by numerical simulations on model corneas. PLoS One.

[31] Li G-Y, Cao Y (2017). Mechanics of ultrasound elastography. Proc. Math. Phys. Eng. Sci..

[32] Touboul D (2014). Supersonic shear wave elastography for the in vivo evaluation of transepithelial corneal collagen cross-linking. Invest. Ophthalmol. Vis. Sci..

[33] Low G, Kruse SA, Lomas DJ (2016). General review of magnetic resonance elastography. World J. Radiol..

[34] Kennedy BF, Wijesinghe P, Sampson DD (2017). The emergence of optical elastography in biomedicine. Nat. Photonics.

[35] Eliasy A (2019). Determination of corneal biomechanical behavior in-vivo for healthy eyes using CorVis ST tonometry: Stress-strain index. Front. Bioeng. Biotechnol..

[36] Curatolo A (2020). Multi-meridian corneal imaging of air-puff induced deformation for improved detection of biomechanical abnormalities. Biomed. Opt. Express.

[37] Maczynska E (2019). Assessment of the influence of viscoelasticity of cornea in animal ex vivo model using air-puff optical coherence tomography and corneal hysteresis. J. Biophotonics.

[38] Singh M (2018). Quantifying the effects of hydration on corneal stiffness with noncontact optical coherence elastography. J. Cataract Refract. Surg..

[39] Lan G, Aglyamov S, Larin KV, Twa MD (2021). In vivo human corneal natural frequency quantification using dynamic optical coherence elastography: Repeatability and reproducibility. J. Biomech..

[40] Kling S (2014). Numerical model of optical coherence tomographic vibrography imaging to estimate corneal biomechanical properties. J. R. Soc. Interface.

[41] Ramier A, Tavakol B, Yun SH (2017). Effect of intraocular pressure on the vibrational resonance of the cornea measured by optical coherence tomography. Invest. Ophthalmol. Vis. Sci..

[42] Silver FH, Shah RG, Benedetto D (2018). Non-invasive and non-destructive determination of corneal and scleral biomechanics using vibrational optical coherence tomography: Preliminary observations. MSA.

[43] Chang EW, Kobler JB, Yun SH (2012). Subnanometer optical coherence tomographic vibrography. Opt. Lett..

[44] Qi W (2013). Resonant acoustic radiation force optical coherence elastography. Appl. Phys. Lett..

[45] Wollensak G, Spoerl E, Seiler T (2003). Riboflavin/ultraviolet-a-induced collagen crosslinking for the treatment of keratoconus. Am. J. Ophthalmol..

[46] Dubois, P., Zemmouri, J., Rouland, J. F., Elena, P. P., Lopes, R., Puech, P. A new method for intra ocular pressure in vivo measurement: First clinical trials. In *2007 29th Annual International Conference of the IEEE Engineering in Medicine and Biology Society* 5762–5765 (Lyon, France, 2007). doi: 10.1109/IEMBS.2007.4353656. 10.1109/IEMBS.2007.4353656 18003322

[47] Coquart L, Depeursinge C, Curnier A, Ohayon R (1992). A fluid-structure interaction problem in biomechanics: Prestressed vibrations of the eye by the finite element method. J. Biomech..

[48] Salimi S, Simon Park S, Freiheit T (2011). Dynamic response of intraocular pressure and biomechanical effects of the eye considering fluid-structure interaction. J. Biomech. Eng..

[49] Kim D (2021). A pilot study for intraocular pressure measurements based on vibroacoustic parameters. Sci. Rep..

[50] Boszczyk A, Kasprzak H, Siedlecki D (2019). Non-contact tonometry using Corvis ST: Analysis of corneal vibrations and their relation with intraocular pressure. J. Opt. Soc. Am. A.

[51] Shah R, Pierce MC, Silver FH (2017). A method for nondestructive mechanical testing of tissues and implants: Nondestructive mechanical testing. J. Biomed. Mater. Res..

[52] Qu Y (2018). Quantified elasticity mapping of retinal layers using synchronized acoustic radiation force optical coherence elastography. Biomed. Opt. Express.

[53] Kennedy BF, Hillman TR, McLaughlin RA, Quirk BC, Sampson DD (2009). In vivo dynamic optical coherence elastography using a ring actuator. Opt. Express.

[54] Schroeder M (1970). Synthesis of low-peak-factor signals and binary sequences with low autocorrelation (Corresp.). IEEE Trans. Inform. Theory.

[55] Adler DC, Huber R, Fujimoto JG (2007). Phase-sensitive optical coherence tomography at up to 370,000 lines per second using buffered Fourier domain mode-locked lasers. Opt. Lett..

[56] Kling, S. Corneal vibrography based on optical coherence tomography (OCT) B-scans. In *Emerging Technologies for Cell and Tissue Characterization* 8, Online Only, (Germany, 2021). doi: 10.1117/12.2615457.

[57] Lan G, Larin KV, Aglyamov S, Twa MD (2020). Characterization of natural frequencies from nanoscale tissue oscillations using dynamic optical coherence elastography. Biomed. Opt. Express.

[58] Han Z (2014). Air puff induced corneal vibrations: Theoretical simulations and clinical observations. J. Refract. Surg..

[59] Keiper DA, Sarin LK, Leopold IH (1965). The vibration tonometer * *From the Franklin Institute and the Wills Eye Hospital. This investigation was supported in part by the National Institutes of Health between 1959 and 1963. It is currently supported by the Pennsylvania Lions Club. Am. J. Ophthalmol..

[60] von Freyberg A (2009). Acoustic tonometry: Feasibility study of a new principle of intraocular pressure measurement. J. Glaucoma.

[61] Zimprich L, Diedrich J, Bleeker A, Schweitzer JA (2020). Corneal hysteresis as a biomarker of glaucoma: Current insights. OPTH.

[62] Blackburn BJ, Jenkins MW, Rollins AM, Dupps WJ (2019). a review of structural and biomechanical changes in the cornea in aging, disease, and photochemical crosslinking. Front. Bioeng. Biotechnol..

[63] Elsheikh A, Joda A, Abass A, Garway-Heath D (2015). Assessment of the ocular response analyzer as an instrument for measurement of intraocular pressure and corneal biomechanics. Curr. Eye Res..

[64] Shah S, Laiquzzaman M, Bhojwani R, Mantry S, Cunliffe I (2007). Assessment of the biomechanical properties of the cornea with the ocular response analyzer in normal and keratoconic eyes. Invest. Ophthalmol. Vis. Sci..

