# Supplementary material for: Co-axial acoustic-based optical coherence vibrometry probe for the quantification of resonance frequency modes in ocular tissue
Source: Sci Rep. 2022 Nov 6;12:18834. doi: 10.1038/s41598-022-21978-8 (PMC9637745; doi:10.1038/s41598-022-21978-8)
Supplement: Supplementary file 1 — Supplementary Information. [file 41598_2022_21978_MOESM1_ESM.pdf]

Co-axial acoustic-based optical coherence vibrometry probe for the quantification of resonance frequency modes in ocular tissue

Ryan McAuley[1], A. Nolan[1], A. Curatolo[2,3,4], S. Alexandrov[1], F. Zvietcovich[2], A. Varea Bejar[2], S. Marcos[2,5], M. Leahy[1], J. S. Birkenfeld[2*]*

[1]Tissue Optics and Microcirculation Imaging Facility, School of Physics, National University of Ireland Galway, Galway, Ireland
[2]Instituto de Óptica, Consejo Superior de Investigaciones Científicas (IO-CSIC), Madrid, Spain
[3]Institute of Physical Chemistry, Polish Academy of Sciences, Warsaw, Poland
[4]International Centre for Translational Eye Research, Warsaw, Poland
[5]Center for Visual Science, The Institute of Optics, Flaum Eye Institute, University of Rochester, New York, USA
[*]Corresponding authors: r.mcauley1@nuigalway.ie, j.birkenfeld@io.cfmac.csic.es

SUPPLEMENTARY INFORMATION

1. Acoustic Signal Synthesis

To induce the sample vibrations, an acoustic signal with a frequency bandwidth containing the expected resonance frequency range is applied to the sample. The acoustic waves are produced by a speaker in response to a voltage signal waveform, created using the following equation describing the linear frequency chirp:

$$V(t) = A\cos\left(2\pi\left(\frac{f_1 - f_0}{2T}t^2 + f_0 t\right)\right) \qquad \text{(S1)}$$

Where: t = the time instance
- $V(t)$ = the instantaneous voltage of the chirp signal at t
- A = the amplitude of the chirp signal
- f_0 = the initial frequency of the chirp signal
- f_1 = the final frequency of the chirp signal
- T = the time taken to sweep from f_0 to f_1

The Fourier Transform of a linear chirp signal suffers from an effect known as Fresnel ripple, as presented in Fig. S1 (b). This is an important implication as when interpreting the frequency content of a signal from a sample, low amplitude peaks/troughs, which are indicative of the resonance modes in the case of the cornea, could be hidden within the oscillations of the Fresnel ripple. If the chirp is used in this way, then resonance modes could be missed or even misidentified which can lead to inaccurate results. To avoid the prevalence of Fresnel ripple in the retrieved chirp signal, a 'flat bandwidth' frequency chirp was generated by a common signal processing technique i.e. shaping of the spectral content in the Fourier domain. This was done by firstly, computing the Fast Fourier Transform (FFT) of the chirp created with Eq.1. and retrieving the magnitude and phase angle for each complex number from the FFT. The below example in Fig. S1 (a) and (b), shows a linear frequency chirp from 0 – 1000 Hz over 1 second at a sampling frequency of 3000 Hz and the absolute and phase values of the FFT of the frequency chirp. Note: the frequency range and sampling frequency were chosen here for illustration purposes.

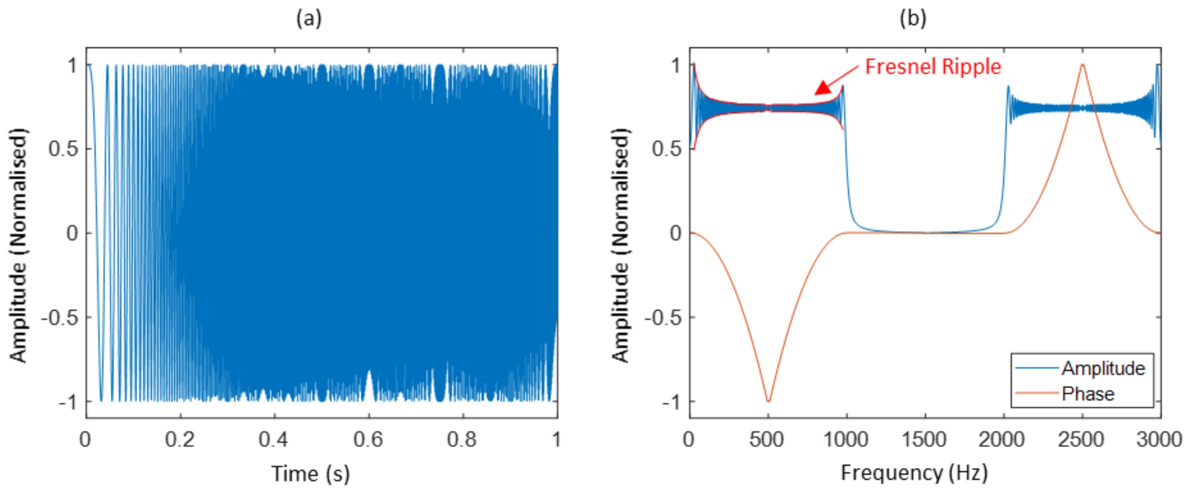

Figure S1. (a) - Time domain linear frequency chirp signal from 0 - 1000 Hz at a sampling frequency of 3000 Hz created using equation (1). (b) - FFT of the signal in (a) showing the frequncy content and phase of the signal in the Frequency domain. Note amplitude and phase values here are normalised. The Envelope of the Fresnel Ripple can be observed in the amplitude spectrum (red).

The spectrum of absolute values presented in Fig. S1 (b) were then discarded and replaced with new values consisting of a normalised flat signal over the 0 – 1000 Hz range, as presented in Fig. S2 (a). The complex numbers from the new absolute and original phase values in Fig. S2 (a), were retrieved from the following formula:

$$ComplexNumber(f) = A(f)\cos\big(\theta(f)\big) + A(f)\sin\big(\theta(f)\big)\, i \qquad \text{(S2)}$$

Where: f = the frequency at which the complex number is to be computed
$A(f)$ = Absolute value at f
$\theta(f)$ = Phase Value at f
i = indicates complex number

The IFFT of the complex values was then computed, resulting in the time domain signal presented in Fig. S2 (b).

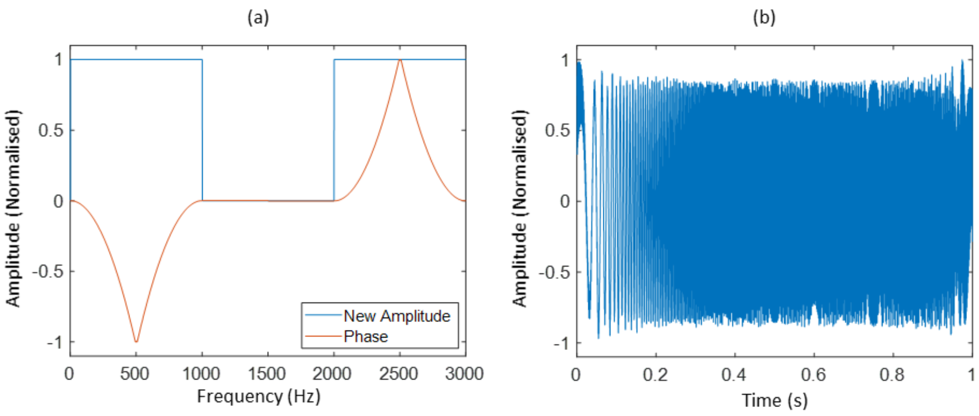

Figure S2. (a) - Frequency domain signal with new flat amplitude spectrum and unchanged phase (normalised). (b) – New Chirp signal created after IFFT of the complex signal created from values from (a) used in equation (2).

This synthesised Linear Frequency Chirp with a flat frequency response was then used in the developed pre-compensation process.

2. – 4. Signal Production, Amplification and Acoustic wave production

The flat bandwidth frequency chirp signal described in the previous section is outputted as a voltage from the computer's audio jack and amplified before reaching the speaker The Audio jack of the computer is connected to the input of the amplifier by a standard auxiliary cable with a 3.5 mm adapter and the amplifier supplies the amplified signal to the speaker via coaxial cables.

4.1 – 4.3. Obtaining frequency content of acoustic waves and spectral reshaping

The flat bandwidth frequency chirp voltage signal from Fig. S2 (b). was supplied to the speaker, playing on a continuous loop. At the same time, a microphone with a known, flat frequency response was used to record the acoustic waves from the speaker. This involves placing the microphone in front of (facing) the speaker at approximately the same position where a sample would be. Fig. S3. (a) below, shows an example acoustic chirp waveform recorded by the microphone from the speaker (modelled using a second order transfer function) and Fig. S3. (b) shows its frequency content after FFT.

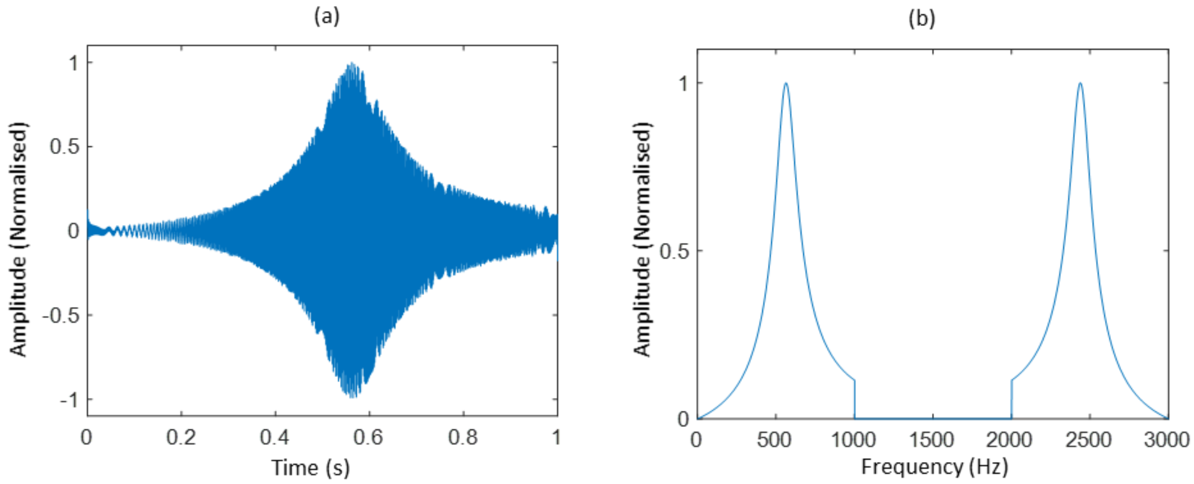

Figure S3. (a) – Modelled Chirp signal from speaker over 1 second with a bandwidth of 1000Hz. (b) – Frequency content of chirp signal.

In Fig. S3. (b), we can see the frequency response of the speaker with a clear resonance at approximately 565Hz. Notice also the lack of Fresnel ripples due to the flat frequency chirp signal and the bandwidth of the signal ending at 1000 Hz. To create a flat acoustic frequency chirp from this speaker, we now introduce ***Pre-compensation***. Essentially, we need to supply the speaker with a linear frequency chirp signal with a frequency response (spectral shape) that is the inverse of the acoustic signal spectrum recorded by the microphone from Fig. S3. (b). To do this, the frequency spectrum absolute values in Fig. S2. (a) are divided by the absolute frequency spectrum in Fig. S3. (b), the phase values remain unchanged and the new frequency chirp signal is reconstructed, as done previously with equation (2). Fig. S4. (a) below shows the new absolute and phase values used to construct the complex numbers and compensated signal and Fig. S4. (b) shows this compensated signal in the time domain.

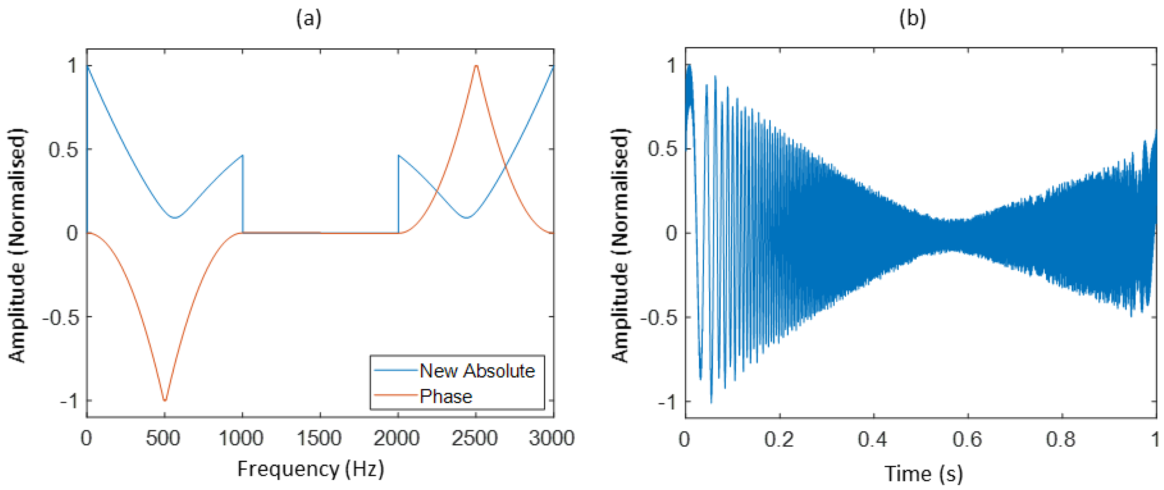

Figure S4. (a) – Phase values from original chirp signal in Fig. S2 (a) and new absolute values which are the result of dividing the absolute values in Fig. S2 (a) by the absolute values in Fig. S3 (b). (b) - New pre-compensated Chirp signal created after IFFT of the complex signal created from values from (a) used in equation (S2).

5. Replacing Microphone with Sample.

The microphone can now be taken away and the sample put in its place. It is important that the surface of the sample e.g. corneal apex, is placed as close to the position of where the microphone was as possible. A convenient way to do this, given the setup, is to use the OCT imaging system to image the microphone during the pre-compensation process and note its position in the OCT image. This image can be then used as a reference to situate the sample as close as possible to the position of the microphone. Once the sample is in position, the pre-compensated frequency chirp can be played on a loop while the OCT system is operating in M-mode over a defined period of time.

IOP vs Resonance Frequency for all CPs with linear fits

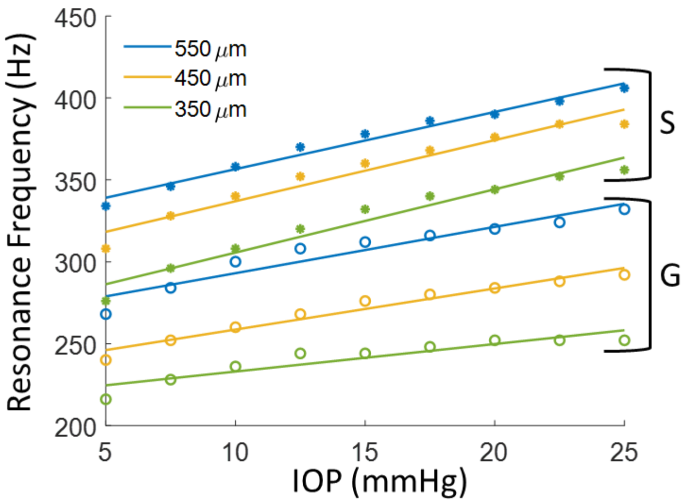

Figure S5. IOP versus resonance frequency for all CPs at IOPs with linear fits added to data.

Thickness vs Resonance Frequency for G and S

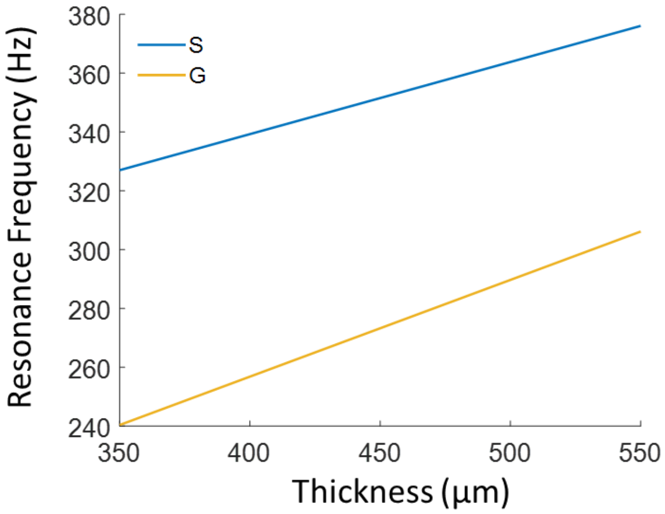

Figure S6. Thickness versus resonance frequency linear fits for G and S, averaged over IOPs.
